# The effect of mebudipine on cardiac function and activity of the myocardial nitric oxide system in ischaemia–reperfusion injury in rats

**DOI:** 10.5830/CVJA-2010-078

**Published:** 2011-11

**Authors:** R Ghyasi, G Sepehri, M Mohammadi, R Badalzadeh, R Badalzadeh, B Rashidi

**Affiliations:** Physiology and Neuroscience Research Centre, Faculty of Medicine, Kerman University of Medical Sciences, Kerman, Iran; Physiology and Neuroscience Research Centre, Faculty of Medicine, Kerman University of Medical Sciences, Kerman, Iran; Department of Physiology, Drug Applied Research Centre, Faculty of Medicine, Tabriz University of Medical Sciences, Iran; Department of Physiology, Drug Applied Research Centre, Faculty of Medicine, Tabriz University of Medical Sciences, Iran; The Young Researchers Club of Tabriz, Islamic Azad University, Tabriz, Iran; Department of Anatomy, Faculty of Medicine, Isfahan University of Medical Sciences, Isfahan, Iran

**Keywords:** ischaemia, nitric oxide, reperfusion, mebudipine, isolated heart

## Abstract

**Objectives:**

Previous studies have suggested that failure of the synthesis of nitric oxide is involved in the pathophysiology of myocardial ischaemia–reperfusion injury. In this study, we investigated the effect of mebudipine, a new dihydropyridine calcium channel blocker, on cardiac function and activity of the myocardial nitric oxide system in ischaemia–reperfusion injury in isolated rat hearts.

**Methods:**

Forty male Wistar rats (250–300 g) were divided into four groups (*n* = 10): sham, control, vehicle and drug groups. The animals were anesthetised with sodium pentobarbital (6 mg/kg intraperitoneal). The hearts were quickly removed, mounted on a Longendorff apparatus and perfused with Krebs-Henseleit solution under constant pressure at 37°C. After 20 min stabilisation period, the ischaemic groups received 30 min global ischaemia and 120 min reperfusion. For the drug and vehicle groups, before ischaemia the hearts were perfused with mebudipine (10^-3^ µM) or ethanol-enriched solution (0.01%) for 25 min, respectively. Myocardial function, and creatine kinase, lactate dehydogenase and total nitric oxide metabolite (nitrite and nitrate) levels were analysed.

**Results:**

Cardiac functions had recovered significantly in the mebudipine group (*p* < 0.01). Furthermore, mebudipine remarkably reduced the levels of lactate dehydogenase and creatine kinase in the coronary effluent and increased myocardial nitric oxide metabolite levels compared with the control group.

**Conclusion:**

Our results indicate that mebudipine reduced the intensity of myocardial ischaemia–reperfusion injury, and that activation of the myocardial nitric oxide system played an important role in this regard.

## Abstract

Early reperfusion is an absolute prerequisite for the survival of ischaemic myocardium. However, reperfusion has been considered a double-edged sword because reperfusion itself may lead to additional accelerated myocardial injury beyond that generated by ischaemia alone. This results in a spectrum of reperfusion-associated pathologies, collectively called reperfusion injury.^1^1 The underlying pathophysiological mechanisms of ischaemia–reperfusion have not been fully elucidated. It has been suggested that an overproduction of oxygen-derived free radicals^2^ and intracellular calcium overload during the first minutes of reflow might be involved.^3^ However, oxygen-derived free radicals and hypercontracture due to calcium overload are not the only candidates responsible for reperfusion injury. Other factors of importance in the pathogenesis of reperfusion injury include platelet- and neutrophil-mediated injury, the renin–angiotensin system and the complement activation.^2^

It is known that nitric oxide (NO) is involved in the regulation of myocardial contractility and contributes to myocardial protection in ischaemic pre- and postconditioning.^4^ NO plays multiple roles in the cardiovascular system, mediating a number of physiological and pathophysiological processes. In smooth muscle cells, NO activates guanylate cyclase by a hem-dependent mechanism, resulting in an increased concentration of guanosine 3′,5′-cyclic monophosphate (cGMP), which leads to a decreased intracellular concentration of Ca^2+^ and subsequent relaxation of the vessels.^5^

Reduced basal availability of NO and impairment of endothelial NO-dependent mechanisms due to dysfunction of the normally protective endothelium may be involved in the pathogenesis of several cardiovascular diseases, including atherosclerosis, hypertension, heart failure, coronary heart disease, arterial thrombotic disorders and stroke.^6^ In cardiomyocytes, the NO/cGMP pathway is involved in the inhibition of Ca^2+^ influx by cGMP-dependent phosphorylation of L-type Ca^2+^ channels,^7^ antagonism of the effects of β-adrenergic stimulation, and decrease in myocardial contractility and heart rate, as well as in reduction in myocardial oxygen consumption and opening of the sarcolemmal K_ATP_ channels. Reduced Ca^2+^ current may alleviate Ca^2+^ overload associated with acute myocardial ischaemia as one of the major mechanisms of ischaemic injury.^5^

Ca^2+^ channel antagonists are used for a variety of diseases, including heart and coronary disease and have become one of the standard first choices of drugs for essential hypertension. They have also become established as therapeutic drugs for angina pectoris, together with β-adrenoceptor antagonists and nitrates.^8^ Ca^2+^ channel antagonists have several features that may relate to myocardial protection during ischaemia and reperfusion. The main effect is reduction in oxygen demand due to a decrease in heart rate and myocardial contractility.^8^ Interference with neutrophil mobilisation and activation may protect against the production of free radicals and the release of proteolytic enzymes.^9^ A direct protective effect may also be produced by interference with ischaemia-induced intracellular Ca^2+^ overload.^10^,^11^

Dihydropyridine Ca^2+^ channel blockers were reported to protect the endothelial function of renal resistance arteries in hypertensive rats^12^ and the mesenteric arteries of rats in circulatory shock.^13^ Endothelial function is important for the preservation of the organ function against ischaemic or hypertensive stress.^14^,^15^ Many studies have reported that Ca^2+^ channel blockers such as amlodipine, nifedipine and benidipine increase NO production.^16^,^17^

Mebudipine is a new calcium channel blocker with a dihydropyridine structure that has a comparable pharmalogical effect while offering some advantages, such as a longer biological half-life to reach peak effect and vasoselectivity.^18^,^19^ There are no reports on the cardioprotective activity of mebudipine and it seems that it may attenuate endothelial dysfunction and increase the production of NO in ischaemic hearts. Therefore, this study was designed to examine the effect of mebudipine on cardiac function and the activity of the myocardial nitric oxide system following ischaemia–repefusion injury in isolated rat hearts.

## Methods

Forty male Wistar rats (250–300 g) were obtained from the laboratory animal house at Tabriz University of Medical Sciences. They were housed in an animal room at 22–24°C and given free access to commercial rat chow and tap water. All the experimental procedures used, as well as rat care and handling were in accordance with guidelines provided by the Experimental Animal Laboratory and approved by the Animal Care and Ethics Committee of the Tabriz University of Medical Sciences. The animals were randomly divided into four groups (*n* = 10): a sham group (without ischaemia), control group (ischaemia without drug), drug group (ischaemia with drug) and vehicle group (ischaemia with ethanol: 0.01%).

## Longendorff protocol

All animals were anaesthetised intraperitoneally with sodium pentobarbital (60 mg/kg) and heparinised with sodium heparin (300 IU intraperitoneally). After opening the chest cavity, the hearts were quickly excised and immersed in ice-cold Krebs-Henseliet (K-H) solution. Then the aortae were cannulated and the hearts were retrogradely perfused via the aorta in a Longendorff apparatus with K-H solution containing (in mM): 118 NaCl, 4.8 KCl, 1.2 MgSO_4_, 1.0 KH_2_PO_4_, 27.2 NaHCO_3_, 10 glucose and 1.25 CaCl_2_. A mixture of 95% O_2_ and 5% CO_2_ was bubbled through the perfusate. A thermostatically controlled water circulator (Satchwell Sunvic, UK) maintained the perfusate and bath temperatures at 37°C.

The hearts were perfused at a constant mean pressure of 75–80 mmHg. During the stabilisation period, a latex balloon (Harvard) attached to the end of a piece of stiff polyethylene tubing was inserted into the left ventricle through the mitral valve. The balloon and tubing were connected to a pressure transducer and filled with normal saline to produce a left ventricular enddiastolic pressure (LVEDP) of 5–10 mmHg at baseline, and the balloon volume was maintained constant throughout the experiment. The LVEDP, LV peak systolic pressure (LVSP) and the peak rates of positive and negative changes in LV pressure (± dp/dt) were measured with a Power Lab System (ADInstruments, Australia). The LV developed pressure (LVDP) was calculated as follows: LVDP = LVSP – LVEDP (mmHg).

The haemodynamic data were recorded continuously on a computer using a Powerlab system. The heart rate (HR) was calculated using a bioelectric amplifier (ADInstruments, Australia) from the electrocardiogram that recorded via two electrodes attached to the apex and the right ventricle of the heart and one reference electrode.

## Ischaemia–reperfusion protocols

The hearts were allowed to equilibrate for 20 min prior to each study. For the ischaemic control group, the hearts were perfused with the K-H solution for 20 min, and then global ischaemia was conducted by interrupting the aortic flow for 30 min, followed by reperfusion with K-H solution for up to 120 min. In the drug and vehicle groups, before ischaemia, the hearts were perfused with mebudipine (0.1 nm) or an ethanol-enriched solution (0.01%) for 25 min, respectively.

Several experimental studies have proven Ca antagonists to be cardioprotective when applied in a concentration that does not produce a negative inotropic or chronotropic effect.^20^-^22^ Mebudipine was therefore applied throughout the study at a concentration of 0.1 nm, which did not cause a negative inotropic or chronotropical effect.

## Biochemical measurements

During the first 10 min of the reperfusion period, the coronary effluent was sampled for lactate dehyrogenase (LDH) and myocardial creatin kinase (CK-MB) measurement. The concentration of LDH and CK in the coronary effluent was measured using related kits (Parsazmoon, Iran) and expressed as units per litre. NO production (nmol/g protein) in the heart homogenates was determined by measuring the total nitrite and nitrate concentration (NO metabolites), using the Griess method.^23^

Deproteinised heart homogenates were used for determination of NO metabolite concentrations (NO_x_). Briefly, 100 μl of supernatant was applied to a microtitre plate well; 100 μl vanadium (III) chloride (8 mg/ml) was added to each well (for reduction of nitrate to nitrite) and this was followed by the addition of the Griess reagents, 50 μl sulfanilamide (2%) and 50 μl N-(1-naphthyl) ethylendiamine dihydrochloride (0.1%). After 30 min incubation at 37°C, the absorbance was read at 540 nm using an ELISA reader (Lab System, Fanland). The concentration of NO_x_ in the heart homogenates was determined from standard linear curves established from 0–150 μmol/l sodium nitrite.

## Statistical analyses

All numerical data are expressed as mean ± SEM. Data on cardiac function were subjected to a two-way analysis of variance (ANOVA), and if statistical significance was established, values were compared using Turkey’s *post hoc* test. The NO_x_ concentrations, and LDH and CK levels were analysed using one-way ANOVA followed by Turkey’s test.

## Results

There were no significant differences in baseline values between all groups (Table 1). In the isolated hearts, when experimental ischaemia was produced by the cessation of coronary perfusion, LVDP and HR rapidly decreased and stopped. A progressive increase in LVEDP was noted in all groups. During the reperfusion periods (10, 30 and 60 min), mebudipine attenuated the increase in LVEDP in the drug-treated group compared with the control group (*p* < 0.01). The administration of mebudipine before ischaemia caused cardiac function to return during the reperfusion period. Mebudipine significantly increased the LVDP and + dp/dt (time = 10, 30 min) (*p* < 0.05), and increased the coronary flow and – dp/dt notably (Table 1).

**Table 1.** Levels Of HR, LVEDP, LVDP, ± DP/DT And CF In Three Groups Of Rats

*Parameter/group*	*Stabilisation*	*Reperfusion*			
		*10 min*	*30 min*	*60 min*	*120 min*
HR (pulse/min)				
Control	284 ± 6.7	20.4 ± 220	243 ± 11.8	233 ± 11.1	212 ± 14.2
Vehicle	275 ± 8.2	19.1 ± 216	256 ± 13.2	247 ± 13.1	227 ± 15.2
Drug	261 ± 13.4	17.3 ± 205	213 ± 19.4	210 ± 19.5	195 ± 18.1
LVEDP (mmHg)				
Control	7.4 ± 0.3	2.5 ± 29.3	27 ± 1.5	23.8 ± 1.2	19.6 ± 1.1
Vehicle	7 ± 0.3	3.1 ± 33.3	31.1 ± 1.1	26.3 ± 0.9	22.5 ± 1.4
Drug	6.7^*^ ± 0.4	1.2** ± 15.5	15.3 ± 1.3**	14.5 ± 1.1*	13.8 ± 1.3
LVDP (mmHg)				
Control	89.8 ± 5.6	42 ± 3.8	45 ± 3.9	45.8 ± 6.7	47.3 ± 4.4
Vehicle	93 ± 7.1	46 ± 5.1	49 ± 4.2	47 ± 7.1	50.4 ± 3.6
Drug	81.6 ± 5.6	69 ± 8**	67 ± 7**	58 ± 5.6	51.8 ± 2.9
+dp/dt (mmHg/s)				
Control	3388 ± 310	1494 ± 468	1575 ± 217.5	1532 ± 189	1445 ± 227
Vehicle	3229 ± 270	1522 ± 259	1590 ± 110	1585 ± 233	1566 ± 210
Drug	3066 ± 336	439* ± 2755	2460 ± 381*	2010 ± 211	1920 ± 200
–dp/dt (mmHg/s)				
Control	1999 ± 217	127 ± 974	1138 ± 149	1054 ± 170	1014 ± 150
Vehicle	1909 ± 264	248 ± 1050	1199 ± 198	1151 ± 109	1106 ± 93
Drug	1863 ± 259	1563 ± 196	1563 ± 196	1343 ± 110	1301 ± 89
CF (ml/min)				
Control	9.8 ± 0.54	7.5 ± 0.18	5.3 ± 0.24	4.5 ± 0.18	3.9 ± 0.27
Vehicle	9.5 ± 0.27	6 ± 0.44	4.9 ± 0.27	4.3 ± 0.16	3.9 ± 0.24
Drug	10.1 ± 0.35	8.2 ± 0.88	6.1 ± 0.37	5.3 ± 0.38	4.6 ± 0.4

Results are expressed as mean ± SEM. for each group (*n* = 10). *p < 0.05 compared with control group and **p < 0.01 compared with control group.

**Fig. 1.**
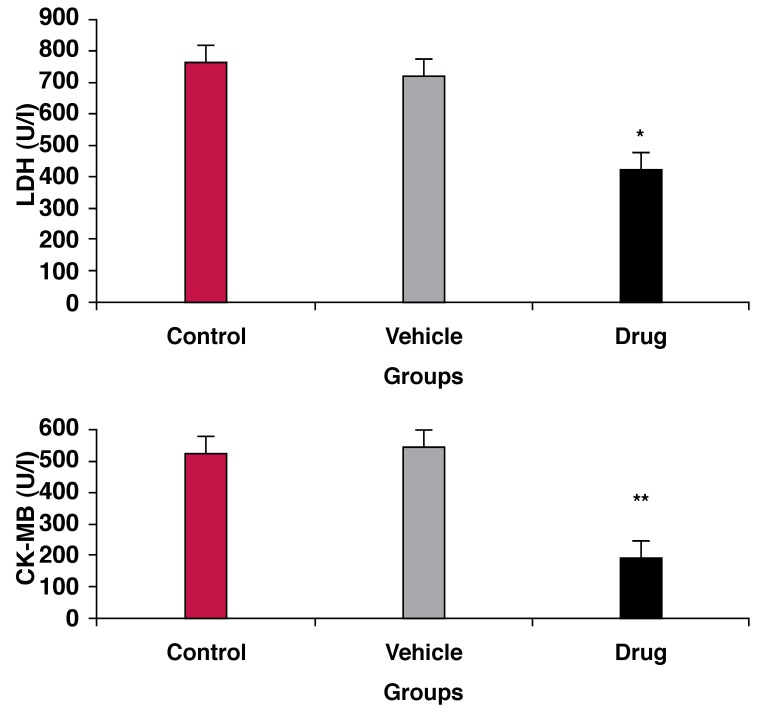
Effect of ischaemia–reperfusion on LDH and CK-MB levels in three groups of rats. **p < 0.01 compared with control group.

**Fig. 2.**
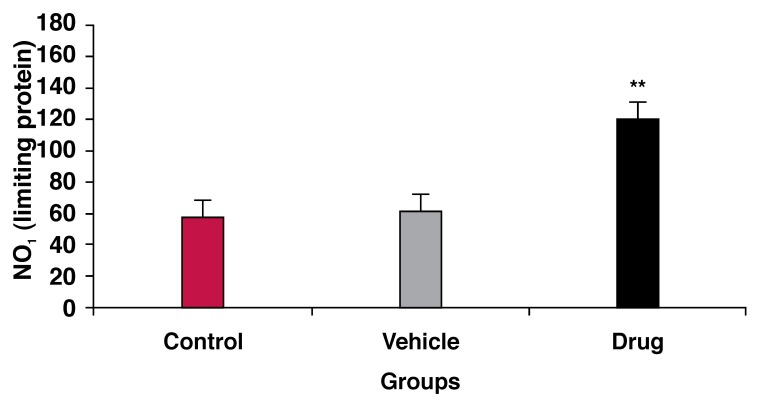
Effect of ischaemia–reperfusion on NO levels in three groups of rats. **p < 0.01 compared with control group.

LDH and CK release in the coronary effluent, as an indicator of cell damage and tissue injury, decreased in the drug-treated group compared with the control group (*p* < 0.01) (Fig. 1). Pretreatment with mebudipine (0.1 nm) increased the concentration of NO metabolites (nitrite and nitrate) in the hearts of the drug-treated group compared with the control group (*p* < 0.01) (Fig. 2).

## Discussion

We examined the influence of mebudipine on myocardial injury resulting from global ischaemia and reperfusion in isolated rat hearts, determined mechanically and biochemically. The findings of this study were that exposure to mebudipine 25 minutes before global ischaemia facilitated the recovery of contractility, decreased LDH and CK levels (indicators of cardiac cellular injury during reperfusion), and attenuated the increase in LVEDP during reperfusion.

Previous studies indicated that mebudipine improved characteristics such as tissue selectivity and significant negative chronotropic effects, and had no noticeable negative effect on the contractility of the heart,^23^ but there have been no studies on the cardioprotective effects of mebudipine against ischaemia–reperfusion injury. This is the first report that a dihydropyridine calcium channel blocker, mebudipine, has the capability of increasing cardiac NO levels in ischaemic hearts, which attenuates the severity of the myocardial ischaemia–reperfusion injury.

It was reported that the other members of this group of drugs, amlodipine, nifedipine and benidipine have cardioprotective effects against myocardial ischaemia–reperfusion injury via NO-dependent mechanisms.^16^,^17^,^24^ Therefore the enhancing effects of NO may not be attributable to the mebudipine, although its capability to increase NO levels due to calcium channel antagonists may be different.

NO is not only produced by endothelial cells,^25^ but also by cardiomyocytes,^26^ erythrocytes,^27^ platelets,^28^ leukocytes and fibroblasts^17^,^29^ in the heart. Several stimuli facilitate NO production. Acetylcholine, bradykinin, purine and norepinephrine stimulate NO synthase.^17^ NO is believed to attenuate the severity of myocardial ischaemia via several mechanisms. NO increases coronary flow, and reduces leukocyte and platelet aggregation.^17^ In our study, the enhancement of coronary flow was notable but not significant, possibly due the concentration of mebudipine that we used. Furthermore, other known physiological effects of NO, such as reduction of ventricular pressure and augmentation of collateral coronary flow^30^ may have contributed to the protective effect of mebudipine against ischemia–reperfusin injury.

In addition, NO may have regulated oxidant-induced alterations in the intracellular Ca^2+^ concentration that caused cytoskeleton derangement, changes in cell shape and ultimately cell necrosis.^31^ In the first minutes of reperfusion, the myocardium may be damaged by the development of contracture (a sustained shortening and stiffening of the myocardium), causing mechanical stiffness, tissue necrosis and the stone-heart phenomenon. Reperfusion-induced contracture can have two different causes, Ca^2+^ overload and depletion of ATP.^1^ Because the volume of the balloon was kept constant during ischaemia and reperfusion in this preparation, an increase in LVEDP reflected an increase in left ventricular wall stiffness or contracture.

Mebudipine significantly attenuated the increase in LVEDP during reperfusion, therefore this drug could decrease cell damage and tissue necrosis. Since this study revealed that mebudipine increased NO levels and reduced LDH and CK release, mebudipine may be effective as a calcium channel antagonist in ischaemic hearts.

## Conclusion

The results of this study confirmed the protective effect of mebudipine against ischaemia–reperfusion injury due to prevention of increased LVEDP, enhanced LVDP and the metabolites of NO, and decreased levels of LDH and CK. Therefore, it may be beneficial for reducing ischemia–reperfusion injuries.
